# Sex-biased avian host use by arbovirus vectors

**DOI:** 10.1098/rsos.140262

**Published:** 2014-11-05

**Authors:** Nathan D. Burkett-Cadena, Andrea M. Bingham, Thomas R. Unnasch

**Affiliations:** Department of Global Health, University of South Florida, Tampa, FL 33612, USA

**Keywords:** sex-bias, infection prevalence, parasite–host interaction, disease ecology

## Abstract

Prevalence of arthropod-borne parasites often differs drastically between host sexes. This sex-related disparity may be related to physiological (primarily hormonal) differences that facilitate or suppress replication of the pathogen in host tissues. Alternately, differences in pathogen prevalence between host sexes may be owing to differential exposure to infected vectors. Here, we report on the use of PCR-based assays recognizing bird sex chromosomes to investigate sex-related patterns of avian host use from field-collected female mosquitoes from Florida, USA. Mosquitoes took more bloodmeals from male birds (64.0% of 308 sexed samples) than female birds (36.0%), deviating significantly from a hypothetical 1:1 sex ratio. In addition, male-biased host use was consistent across mosquito species (*Culex erraticus* (64.4%); *Culex nigripalpus* (61.0%) and *Culiseta melanura* (64.9%)). Our findings support the hypothesis that sex-biased exposure to vector-borne pathogens contributes to disparities in parasite/pathogen prevalence between the sexes. While few studies have yet to investigate sex-biased host use by mosquitoes, the methods used here could be applied to a variety of mosquito-borne disease systems, including those that affect health of humans, domestic animals and wildlife. Understanding the mechanisms that drive sex-based disparities in host use may lead to novel strategies for interrupting pathogen/parasite transmission.

## 2. Introduction

Sex-biased prevalence of mosquito-borne pathogens/parasites is an important theme in disease ecology. Field evidence demonstrates that males and females of a host species often differ considerably with respect to infection prevalence. Depending on the host taxon and the parasite/pathogen, various relationships between host sex and vector-borne agents have been reported in natural populations. Male lizards in Puerto Rico, for example, were found to have significantly higher prevalence of saurian malaria (*Plasmodium* spp.) than females (32% of 3296 males, versus 22% of 1439 females) [1]. A meta-analysis exploring sex-biased parasitism of avian hosts by a variety of vector-borne blood-parasites (*Haemoproteus*, *Leucocytozoon*, *Trypanosoma* and *Plasmodium*) found that female birds were infected significantly more frequently than males, especially with respect to *Haemoproteus* spp. parasites [2].

Hypotheses explaining the occurrence of sex biases of mosquito-borne pathogens/parasites mainly fall into two categories. The first category invokes physiological differences between the sexes to explain how parasites/pathogens may replicate more successfully in one sex, leading to differences in disease prevalence between the sexes [3]. Female wild house sparrow (*Passer domesticus*) experimentally infected with *Plasmodium relictum*, a mosquito-transmitted hemoparasite [4], had longer lasting patent infections with greater incidence than did male birds. Fifty per cent of inoculated female sparrows had patent infections, while far fewer inoculated males (28.5%) demonstrated patent infections upon recapture [4]. In laboratory studies, male hamsters experimentally infected with *Leishmania* spp. parasites (transmitted by blood-feeding sand flies (Diptera: Psychodidae) in nature) had significantly larger and more severe cutaneous lesions and greater parasite burdens than did experimentally infected females [3]. Females with artificially elevated testosterone levels had significantly larger lesions than females with natural testosterone levels, suggesting that this gender-related difference was caused, in part, by hormonal differences between the two genders [3]. In general, the complex interactions between the parasite and the host animal's immuno-endocrine system are thought to produce differential levels of parasite prevalence in wild populations [5].

The second category of hypotheses ascribes sex biases in prevalence of vector-borne infections as an effect of unequal exposure [6,7], i.e. differences in contact with vectors, modulated by behavioural or morphological differences between sexes. Behavioural or morphological differences between genders are thought to be the mechanism modulating vector–host contact, thereby regulating rates of exposure between the sexes. Breeding males of many species engage in ‘risky’ behaviours, such as ritualized display and territorial battles, that often place them at increased risk of predation [8] and can also increase their exposure to blood-feeding arthropods [9]. Male tungara frogs, for example, vocalize to attract mates. Blood-feeding (*Corethrella* spp.) flies eavesdrop on the calls to locate and feed upon male frogs [10], resulting in high prevalence of blood-parasites (*Trypanosoma* spp.) in males (more than 70%), while natural infections in females are unknown [11]. This extreme example illustrates how different behaviours of males and females affect their contact rates with vectors, culminating in differential infection prevalence.

Birds are critical hosts in the amplification, dissemination and maintenance of several mosquito-borne viruses that affect humans, including West Nile virus (WNV), St Louis encephalitis virus and eastern equine encephalitis virus [12]. Therefore, understanding factors that influence patterns of host use in birds is a matter of practical importance. In this study, we employ a PCR-based technique to identify the sex of avian hosts from mosquito bloodmeals and explore patterns of avian host use, as it relates to host sex.

## 3. Material and methods

Mosquitoes were collected weekly from forested wetland habitat spanning 12.2 km of the Hillsborough River drainage, near Tampa, FL, USA, during the two consecutive winters (December to February) of 2012 and 2013. Hand-held aspirators were used to collect female mosquitoes from 18 artificial resting shelters [13] scattered throughout the habitat according to the methods described in [14].

Blood-engorged female mosquitoes were assayed using PCR to identify the source species of the vertebrate host blood. DNA extraction and nested PCR amplification of a portion of the cytochrome *b* gene (383 and 296 bp) were conducted as previously described [15]. Amplicons were purified, and then sent for sequencing (Eurofins MWG Operon, Huntsville, AL, USA). Sequences with greater than or equal to 95% match to GenBank (NCBI BLAST) sequences were interpreted as correctly identified host species. We then assayed avian-derived bloodmeal samples by PCR to determine sex of the host using primers targeting the highly conserved [16–18] chromo-helicase-DNA-binding protein (CHD) gene on the W and Z chromosomes [19]. Reaction conditions matched published protocols [19]. Sex of the bloodmeal sample was determined by electrophoresis of PCR product on 2% agarose gel in Tris-acetate-EDTA buffer, stained with ethidium bromide. The PCR assay produces two fragments of different sizes in females (heterogametic sex) and one fragment in males [20]. Protocols were validated via blind tests using blood samples from captive zebra finches of known sex from the University of South Florida, College of Medicine Zebra Finch Colony (IACUC no. IS00000396).

The *χ*^2^-test was used to test whether the sex-related host use (proportion of meals from each sex) differed among bird-biting mosquito species (more than 20 bird bloodmeals). *χ*^2^ goodness of fit test was used to determine whether bloodmeals originating from males and females deviated significantly from a hypothetical 1 : 1 sex ratio [21].

## 4. Results

**Figure 1.**
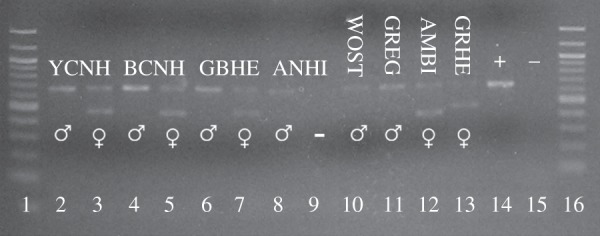
Agarose gel stained with ethidium bromide showing sex determination of avian hosts from field-captured, blood-fed mosquitoes. Lanes: 1 and 16, ladder; 14, positive control; 15, negative control; YCNH, yellow-crowned night heron; BCNH, black-crowned night heron; GBHE, great blue heron; ANHI, anhinga; WOST, wood stork; GREG, great egret; AMBI, American bittern; GRHE, green heron.

Three mosquito species (*Culex erraticus*, *Culex nigripalpus* and *Culiseta melanura*) yielded a sample size of over 20 bird bloodmeals (table 1). Other species fed primarily upon non-avian hosts (mammals, reptiles and amphibians) (table 1). Sex determination was successful from 77.4% (range 66.6–88.0% by mosquito species) of the total avian bloodmeals from the field-captured mosquitoes (table 1; figure 1).

**Table 1.** Host use and sex determination of female mosquitoes from field sites in Florida, USA (2011–2013) determined through PCR assays targeting the cytochrome *b* gene (host species) and CHD gene (avian sex).

	total meals^a^	avian-derived meals		avian sex determined		avian sex negative^b^	
mosquito	*N*	*N*	%	*N*	%	*N*	%
*Anopheles crucians*	22	3	13.6	3	100.0	0	0.0
*Anopheles perplexens*	7	1	14.3	0	0.0	1	100.0
*Anopheles quadrimaculatus*	34	3	8.8	2	66.7	1	33.3
*Culiseta melanura*	60	52	86.7	37	71.2	15	28.8
*Culex erraticus*	500	300	60.0	236	78.7	64	21.3
*Culex nigripalpus*	40	29	72.5	23	79.3	6	20.7
*Culex peccator*	7	5	71.4	4	80.0	1	20.0
*Culex salinarius*	1	1	100.0	1	100.0	0	0.0
*Culex territans*	31	2	6.5	1	50.0	1	50.0
*Uranotaenia sapphirina*	2	2	100.0	1	50.0	1	50.0

^a^Includes all identified feedings (birds, mammals, reptiles and amphibians).

^b^Sex could not be determined, based on the procedures used (no amplification).

**Figure 2.**
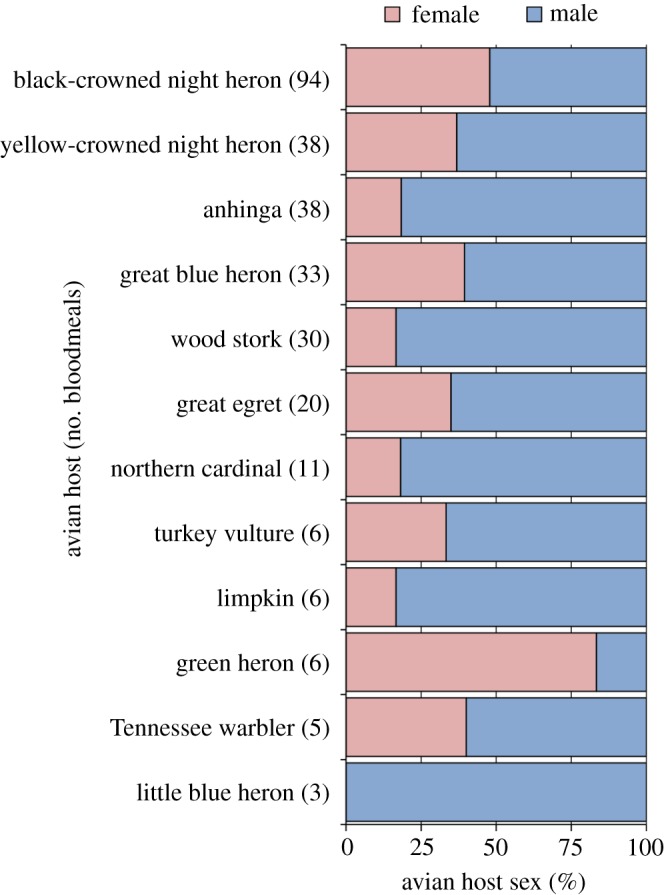
Sex-biased avian host use by mosquitoes from wetlands in Hillsborough County, FL, USA, December–February 2012–2013.

Overall, 64.0% of sexed bloodmeals were from male birds, a significant deviation from a hypothetical 1 : 1 ratio (χ_1_^2^ = 24.01; *p*<0.001). Male-biased host use was observed across bird species (figure 2), with one exception (1/6 green heron meals from males). Mosquitoes took 80–85% of bloodmeals from males of limpkin (5/6), northern cardinal (9/11), wood stork (25/30) and anhinga (31/38); 60–70% from males of turkey vulture (4/6), great egret (13/20), yellow-crowned night heron (24/38), great blue heron (20/33) and tennessee warbler (3/5); and just over half (49/94) from black-crowned night heron males. Sex-related host use (proportion of meals from male and female birds) did not differ significantly among bird-feeding mosquito species (χ_2_^2^ = 0.122; *p*=0.94). *Culex erraticus*, *Cx. nigripalpus* and *Cs. melanura* took 64.4, 60.9 and 64.9% of bloodmeals from male birds, respectively (table 2).

**Table 2.** Gender (F, female, M, male) of avian hosts from three mosquito species (*Culiseta melanura*, *Culex erraticus* and *Culex nigripalpus*) from field sites in Florida, USA, as determined by PCR.

		*Culiseta melanura*		*Culex erraticus*		*Culex nigripalpus*		total	
bird	total	F	M	F	M	F	M	F	M
American bittern (*Botaurus lentiginosus*)	2	1	0	0	1	0	0	1	1
anhinga (*Anhinga anhinga*)	38	0	1	5	27	2	3	7	31
black-crowned night heron (*Nycticorax nycticorax*)	90	5	3	38	43	1	0	44	46
great blue heron (*Ardea herodias*)	28	0	1	9	14	1	3	10	18
great egret (*Ardea alba*)	20	0	0	7	13	0	0	7	13
green heron (*Butorides virescens*)	6	1	1	4	0	0	0	5	1
limpkin (*Aramus guarauna*)	6	0	0	1	5	0	0	1	5
little blue heron (*Egretta caerulea*)	3	0	0	0	0	0	3	0	3
Muscovy duck (*Cairina moschata*)	2	0	0	1	1	0	0	1	1
northern cardinal (*Cardinalis cardinalis*)	10	2	8	0	0	0	0	2	8
pied-billed grebe (*Podilymbus podiceps*)	2	0	0	2	0	0	0	2	0
Tennessee warbler (*Vermivora peregrina*)	5	2	2	0	1	0	0	2	3
turkey vulture (*Cathartes aura*)	6	0	1	0	1	2	2	2	4
wood stork (*Mycteria americana*)	29	0	2	4	21	1	1	5	24
yellow-crowned night heron (*Nyctanassa violacea*)	37	1	1	12	23	0	0	13	24
others^a^	12	4	1	2	2	2	4	4	8

^a^Single individuals of black vulture, *Coragyps atratus* (male); Carolina wren (male); great horned owl, *Strix varia* (male); hermit thrush (male); house wren, *Troglodytes aedon* (male); loggerhead shrike, *Lanius ludovicianus* (male); mourning dove, *Zenaida macroura* (female); osprey, *Pandion haliaetus* (male); tufted titmouse, *Baeolophus bicolor* (female); white ibis, *Eudocimus albus* (female); wild turkey, *Meleagris gallopavo* (male); Wilson's snipe, *Gallinago delicata* (female).

## 5. Discussion

Of sexed samples, nearly twice as many bloodmeals were from male as female birds, a significant deviation from a 1 : 1 ratio. Given the difficulties in sexing birds based on external morphology [22,23], we can only speculate on how sex ratios from the mosquito bloodmeals compares to that of the natural avian populations at our field sites. While adult sex ratios are undocumented for many bird species, a comprehensive review found that male-biased adult sex ratios are more common than balanced or female-biased sex ratios [23]. In wading birds (herons, egrets and allies), the most common hosts in this work (table 2 and figure 2), male-biased adult sex ratios are more than twice as common as female-biased adult sex ratios [23], so it is possible that the male-biased sex ratios from mosquito bloodmeals is representative of the natural adult sex ratios. Skewed adult sex ratios in birds are thought to be a result of unequal mortality, particularly for nesting females, as opposed to genetically skewed sex ratios in offspring [23].

That sex-related host use did not differ significantly among mosquito species is supportive of the idea that broad patterns of host use are driven more by traits of the host animal than by the mosquito, as indicated from recent field studies of mosquito host use of confined birds of prey [24]. The three mosquito species investigated for sex-biased host use are notable vectors of arboviruses for which birds are primary reservoir hosts. *Culex erraticus* and *Cs. melanura* are epizootic and enzootic vectors of eastern equine encephalomyelitis virus [25,26], respectively, while *Cx. nigripalpus* is the vector of St Louis encephalitis virus [27,28].

The methods employed here permitted sex determination of 77.4% of samples, similar to that of molecular sexing from skin samples from museum specimens (approx. 75%) [29], but substantially lower than that from fresh tissue samples (generally 100%) [29]. This lower percentage of successful sexing is probably owing to partial digestion in the mosquito midgut.

Adult sex ratios of birds often differ between seasons [23], so the male-biased host use observed here (winter) may not persist into the breeding season. Support for seasonal differences in sex-biased host use may be inferred from results of recent work from the northeastern USA, where *Culex restuans*, suspected enzootic vector of WNV, was found to take more bloodmeals from female birds during the nesting season [21]. The biased feeding upon females was linked to greater susceptibility of brooding female birds to attacking mosquitoes [21], as previously demonstrated [30]. In addition, bird species that have balanced sex ratios during the nesting period may have dramatically unbalanced sex ratios during other parts of the year, particularly winter [23], owing to partial migration of the population or differential mortality between sexes. The most commonly fed-upon species in this study are primarily residents, although they may not breed at their overwintering sites, complicating predictions.

The use of genetic markers (DNA ‘fingerprinting’) to identify sex of human hosts from field-captured blood-engorged mosquitoes has previously been used to investigate whether sex-biased feeding upon humans (among other variables) drives sexual disparities in dengue virus infection [31,32]. DNA fingerprinting requires development of a large microsatellite database for comparing unknown field samples, which represents a very substantial effort, even in a relatively small community. Assays that target the sex chromosome do not require a comparative database, and could be used to explore sex-linked differences in other human and wildlife diseases. For example, prevalence of human *falciparum* malaria can be five times higher in adult males than females [33]. If this disparity in infection prevalence is related to sex-biased host use, the methods outlined here could be used to determine whether contact rates with malaria vectors drives the observed epidemiological patterns. This might, in turn, provide new insights into how public health programmes might shift their strategies to interruption transmission of human pathogens.

## Data accessibility

Raw data available at Dryad: doi:10.5061/dryad.tj54k.
